# Data Completeness and Concordance in the FeverApp Registry: Comparative Study

**DOI:** 10.2196/35510

**Published:** 2022-11-02

**Authors:** Larisa Rathjens, Ingo Fingerhut, David Martin, Sara Hamideh Kerdar, Moritz Gwiasda, Silke Schwarz, Ekkehart Jenetzky

**Affiliations:** 1 Faculty of Health/School of Medicine Witten/Herdecke University Witten Germany; 2 Pediatric Office Kleiner Piks Bochum Germany; 3 Department of Pediatrics Eberhard-Karls University Tübingen Germany; 4 Department of Child and Adolescent Psychiatry and Psychotherapy University Medical Center of the Johannes-Gutenberg-University Mainz Germany

**Keywords:** registry, data quality, completeness, concordance, ecological momentary assessment

## Abstract

**Background:**

The FeverApp registry uses ecological momentary assessment (EMA) to collect parental data on pediatric fever for scientific research. The mobile app FeverApp educates parents on safe fever management and serves as a fever diary.

**Objective:**

The focus of this study was to evaluate the completeness and concordance of the EMA-based FeverApp registry with regard to its data quality from a multilevel perspective.

**Methods:**

Structured descriptions of fever episodes by health care professionals from an office were used as reference. The number of children, their sociodemographic data, and agreement of fever episodes, with maximum temperature, intake of antipyretics and antibiotics, and physician visits, were compared with the entries in the corresponding physician’s reference records. The data quality indicators for completeness, meaning the extent to which the necessary data for the registry has actually been submitted, and concordance, which is the correspondence of the value of a data element with a reference source, were chosen to analyze whether EMA may be a suitable method for this kind of registry.

**Results:**

In both data sources, 1012 children were available for comparison over 16 months. The completeness of gender (1012/1012, 100%) and date of birth (1004/1012, 99.2%) information was high, and the mismatches were 0.69% (7/1012) and 1.19% (12/1012), respectively, between the sources. Of these 1012 children, 668 (66%) registered fever episodes in FeverApp. They relate to 534 families with 953 fever episodes in the reference records and 1452 episodes in the FeverApp registry. Of the 534 families, 183 (34.3%) refrained from visiting the office during fever episodes but nevertheless documented them in FeverApp. Largest part (766/1452, 52.75%) episodes were recorded exclusively in the FeverApp registry by 371 (371/534, 69.5%) families. The remaining 686 (47.2%) episodes of 391 (58.5%) children from 351 (65.7%) families were comparable with the reference data source in terms of physician visits, medication, and temperature. The completeness ranged, depending on the kind of variable, from 11.5% to 65% in the registry and from 7.6% to 42.6% in the office. The 953 fever episodes reported by the reference office consisted of 681 (71.5%) acute and 272 (28.5%) past episodes. In FeverApp, most past (262/272, 96.3%) but less acute (424/681, 62.3%) episodes have been entered. The concordance rates were varied: 90.2% for antibiotic use, 66.6% for antipyretic use, 61.7% for physician visits, and 16% for the highest temperature during the fever episode.

**Conclusions:**

Both sources delivered only partial data, and the rates of completeness and concordance depended on the kind of variable. However, the FeverApp registry showed higher documentation and precision rates than professional records for all considered variables. Therefore, EMA may play a unique supplement for research in ambulatory care. FeverApp could support pediatric offices, especially during the pandemic.

## Introduction

### Background

Modern technologies enable registry studies via mobile phone apps through ecological momentary assessment (EMA) [1,2]. On the one hand, this is beneficial because of straightforward data collection: the study participants enter the data themselves, saving time and costs for study personnel. In sudden symptoms, for example, fever, ecological observation during long periods is more applicable in contrast to paper-based protocols [3] in other study situations. On the other hand, the quality of the entered data is not controlled separately. Transfer errors in paper-based documentation are reduced, and immediate plausibility checks are possible. It has yet to be proven to what extent they are comparable with the data from medical personnel, a common standard in registries. Especially, if the real-time data of nonprofessionals are used as registry data, their comparability and difference should be monitored specifically, at least in samples.

On-site monitoring and source data verification are important methods to improve data quality not only in clinical studies, but also in other medical research contexts. In an app-based, real-time registry, there are usually no further sources. Medical registries often rely on medical professionals. This is useful for diseases, but symptoms such as fever are often acknowledged or recorded by nonprofessionals. A further challenge is that health care routine data are often not appropriately structured. Comparable structured data from health care professionals are needed to verify the quality of app-based registry data generated by parents. An example of such a registry that relies on parental real-time EMA is the FeverApp registry.

### FeverApp Registry

The Federal Ministry of Education and Research in Germany has funded 6 model registries in 2019 [4]. They should provide exemplary features of registries, such as the consideration of observing (parent using an app) and observed (children) units at suddenly occurring events (fever episodes) [5]. The registry protocol was published [1] and registered in the German Clinical Trials Register with the registration number DRKS00016591.

In general, FeverApp could be used completely anonymously if no identifying entries are made. There are currently no mandatory fields that force identification. The app is freely accessible, but users need an access code from a pediatric office that generates a random family code. This random pseudonym could nevertheless identify if it is made public. Hence, the family code gives the opportunity to share access to further family members. This procedure ensures the acknowledgment of the treating physician, even if no reference records with direct recording of the family code were made by the participating offices.

The FeverApp registry collects data via parental EMA of the child’s febrile episodes since September 2019. Recruitment was started in a large pediatric reference office. Since July 2020, FeverApp has spread on a larger scale to multiple pediatric offices. Until now, pediatric offices have solely granted access to parents.

FeverApp is a mobile app in which parents and caregivers can record, track, and manage children’s fever episodes and symptoms. By providing scientific information based on current guidelines [6], FeverApp helps parents to understand fever better and manage it safely and comfortably. The goal of FeverApp is to establish a model registry through the self-documentation of fever management by families, thereby drawing conclusions about the implementation of the guidelines. It aims to inform parents that fever is not a disease but rather a symptom of the immune defense system fighting the underlying causes [7-9]. To strengthen the immune system, the intake of antipyretics and antibiotics should be restrained. It also educates parents that the use of health care resources depends on the child’s age, emphasizing that these are not mandatory unless specific warning signs are observed. In this case, a physician’s visit should be considered. Therefore, in case of solely high temperature, an immediate visit to a physician or medication is not recommended.

The submitted entries and interactions between different pages of the app are stored locally in the app within an open-source JavaScript database, PouchDB 7.3.0, which synchronizes it with Apache CouchDB 2.3.1 when connected to the internet. The latter database is centrally located on the University of Witten/Herdecke servers, and the documents of CouchDB are transformed and transferred daily to MongoDB. Several relational data tables are exported in CSV format, extracted on demand through SQL scripts, and processed in SPSS (version 27; IBM Corp). These data represent the FeverApp registry [5].

There are specific access codes for test purposes to ensure that, routinely, only real observation data are collected. To consider a high standard of data correctness and security, all decentral data deleted from the app are also deleted from the central registry. If a parent deletes any data on their mobile phone, this deletion is synchronized with the central CoachDB, and the data are no longer available for export. Therefore, wrong entries can be reduced.

The aforementioned 6 registries agreed to compare their data quality but could not agree on a common understanding of completeness because of the different scope of each registry. Furthermore, the funding reviewer questioned whether reliable data could be collected via a parental app.

### Aim of the Study

Therefore, this study aimed to evaluate 2 important indicators for trueness: completeness and concordance. It especially takes into account the multilevel or clustered structure of the collected EMA-based data.

## Methods

### Conception of Data Quality

There are different approaches to conceptualize data quality. Weiskopf and Weng [10] categorized 5 dimensions of data quality in their review of the clinical research literature discussing data quality assessment methodology for electronic health record (EHR) data. These are completeness, correctness, concordance, plausibility, and currency. The approaches used for data quality assessment are summarized as follows: comparison with gold standards, data element agreement, data source agreement, distribution comparison, validity checks, log review, and element presence. The authors conclude that there is little consistency or potential generalizability in the methods used to assess data quality in EHRs, and they demand for systematic methods of EHR data quality assessment.

Kahn et al [11] proposed a conceptual model for data quality assessment in EHR data that can improve data utility over time. The framework was created especially for clinical research. This concept is followed by the approaches of Weiskopf et al [12] and Lee et al [13]. All authors underline that quality assessment should be customized for every single study. This statement raises the question of how the data quality of EMA-based registry studies should be realized.

This gap is closed by the concept of adaptive management of data quality: *The Technology and Methodology Platform for Networked Methodological Medical Research* (TMF) published an approach for the independent assessment of data quality and its improvement in 2006. The manual *Guidelines for the Adaptive Management of Data Quality for Cohort Studies and Registers* (GAMOQ) [14] enables the evaluation of the quality of data concerning different aspects. The novel approach of these guidelines is the distinction of the data quality conception into 3 dimensions. It allows assessing data quality in a structured manner and has become a standard approach in Germany [15-18]. It is crucial to ensure that the collected data in an app-based registry are of high quality in terms of their structures, processes, and outcomes (to aspects of health data quality).

According to the recommendations of the GAMOQ, data quality assessment can be divided into 3 dimensions: data integrity, data organization, and data trueness. These correspond to the approaches developed by Donabedian [19] for the assessment of the quality of medical data: structure (ie, integrity of data), process (ie, organization of data), and outcome quality (ie, trueness of data). Each of these data quality aspects can be described with specific data quality indicators (DQIs). The GAMOQ includes a total of 51 DQIs. The choice of suitable DQIs for the quality assessment of data integrity, organization, and correctness depends on the specific study situation. Thus, the GAMOQ offers a flexible tool for the systematic evaluation of the quality of registry data. Defined threshold values for DQIs are a prerequisite for calculating the overall score for data quality from the individual indicator values.

An important quality indicator for external validity or representativeness is the completeness (confer in the GAMOQ as TMF-1042) of the collected data elements. This quality indicator describes the trueness of data. Concordance (confer in the GAMOQ as TMF-1002) is one of the DQIs that is used for the description of the integrity of data [14].

### Completeness

The DQI *completeness* is defined as the extent to which the necessary data that could be included in the registry have been submitted. Other registries or patient records in medical offices are possible data sources for determining the necessary data, which could be included in the registry. Nonnemacher et al [14] underlined that an examination of the data quality in registries is mostly done by comparison with other data sources.

In this innovative parent-based and app-based registry, technical and informative mandatory fields have to be distinguished. FeverApp keeps nearly all fields as technical voluntary fields, although they are informative mandatory. Therefore, if a technical voluntary field would not be understood as being incomplete, then no field could become informative incomplete. As FeverApp is a model registry, we apply DQIs as informative mandatory fields, although they are technical voluntary. This is a special feature of this model registry. Completeness is analyzed as informative mandatory.

### Concordance

Concordance is defined as the correspondence of the value of a data element with a reference source. Concordance is usually used as a DQI for data structure [14], but it can also be regarded as a DQI for the completeness of data [20]. As an alternative for *completeness*, it would be possible to use the DQI *concordance* under awareness that the physician’s registry could be seen as the gold standard for the data quality of EMA. It is common that fever events are recorded in pediatrician offices. However, it is usually not done in a structured way, as we have done it. In this pediatric office, each patient was asked regarding fever. In the app-based registry, this was a voluntary commitment. Hence, these structured office records may be seen as the gold standard.

Usually, the threshold value for the concordance rate and completeness rate is defined as 95% for registries by medical professionals [14]. Thresholds are not scientifically validated and can be changed with justification [16].

### Multilevel Perspective

**Figure 1:**
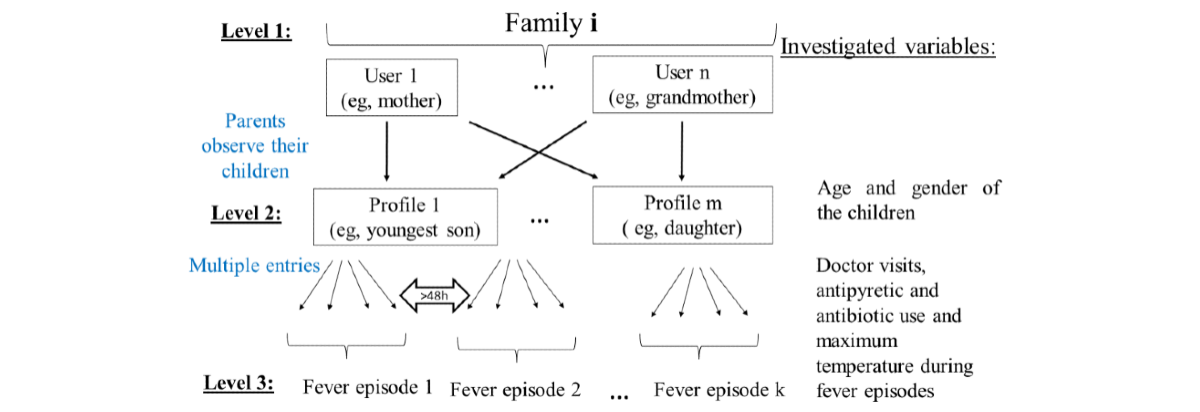
Multilevel observation units of the FeverApp registry.

FeverApp is a tool for parents to observe the fever episodes of their children. A fever episode is defined by a series of multiple entries without cessation for >48 hours. It always relates to a child profile, which belongs to a family, so this can be considered a cluster. If several users install FeverApp with the same family code, they will share the same profiles of their children. Hence, a family is the major observation unit of the registry. This contrasts with the reference records of a pediatric office, where a child and an adolescent is the observation unit and not the total family. However, the main point of interest is the fever rather than the family or children. For children, who we label as profiles, we can consider some sociodemographic data (date of birth and gender) for comparison. Because each child within a family can have several fever episodes, each consisting of multiple entries and different variables during a period, these could be considered as a further level. Owing to these circumstances, any reporting of quality indicators, such as completeness or concordance, depends on the considered observation object. We have illustrated this structure in Figure 1.

The collected EMA data rely on event-based sampling at the family level and on time-based sampling at the fever-episode level: children have fever occasionally, but researchers intend to monitor how body temperature and other indicators, such as parental confidence and children’s well-being, vary over time during a fever episode. The EMA design was combined [21] owing to the multidimensionality of the data.

We are aware that with additional offices, further levels such as physicians’ offices and regions or countries could be integrated above the family level, and pointing downward, single entries and the aspects of fever episodes may be considered separately. The schematic figure (Figure 1) depicts the data structure of the central FeverApp registry where “i” denotes an arbitrary number of the family (the possible app user values are from 1 to n, profile values from 1 to m, and fever episodes from 1 to k, wherein n, m, and k are any natural numbers). Participating pediatric offices in the country (currently only German-speaking countries) distribute an access code for the app to several families with children that are interested in using FeverApp. The access code of the pediatric office generates a random family code, which can be shared with other family members to access the same profiles of the children. The random family code is an 8-character lowercase combination and uniquely defines the participating family.

Hence, participating families (level 1) are the observation units, defined by a family code and related to a pediatric office. In each family, there can be several users, that is, app installations with the same family code, who observe the same children (profiles). Therefore, ≥1 users of a family document ≥1 profiles (level 2) with ≥1 entries in FeverApp. These entries document ≥1 fever episodes (level 3), which are currently defined until a child is marked as *healthy*. Some long fever episodes may have been recorded erroneously when the users forgot to click the *child healthy* button, which naturally defines the end of an episode. Therefore, episodes were redefined using the definition of fever duration. If no entry was made for at least 2 days (>48 h), the next entry is regarded as a new fever episode. The time of the entries is recorded, but if the entries are made retrospectively, for example, after the end of a night, the user is called to enter the time of real occurrence to be used for calculations. As fever occurs especially at an early age, the project primarily intends to collect data about children who are yet to reach adulthood. Since October 2020 (app version 1.7), it is possible to enter a separate physician’s office for each profile. In this case, the pediatric office can differ between profiles and from the distribution office for each family [5,22].

As part of this model registry, we established structured reference records regarding fever in a pediatric physician’s office, which may be considered as true to assess the quality indicator completeness.

### Physician’s Reference Records

One large pediatric office in Bochum (North Rhine-Westphalia, Germany) has participated in the FeverApp registry study since it was established in September 2019. This reference office very accurately documents several fever-related questions [Multimedia Appendix 1] for each child in the physician’s reference records with separate fields in the EHR system Medistar from the CompuGroup. Each family participated with a written informed consent for the comparison of registry data with the reference records of their children. The main purpose of this effort was to validate the parental FeverApp registry data. From the EHR system, these data were extracted using an SQL export. These reference record data from the physician’s office could be considered a second registry to validate the parental FeverApp registry.

The records in the pediatric office contain the following information about a patient’s fever episodes: date of the visit, past and acute fever episodes, fever duration, maximum temperature level, and medication. A past fever episode is fever that is only reported to the physician when they asked regarding any fever episode since the last visit. An acute fever episode is defined as any visit to the physician with a child having acute fever. It was noted whether children received any antipyretics and antibiotics including their names. The FeverApp access code that families have received is registered in the EHR and serves as an identifier. These parents should also answer whether they actually used FeverApp during the reported fever episodes. As sociodemographic information, only the date of birth and gender were considered for each patient.

### Statistics

Data analysis was performed using the statistical software R 3.6.3 [23], and data visualization was performed with the R-package ggplot2 [24]. The ratios for concordance are calculated with the number of matches in relation to the number of possible matches. Whereas Nonnemacher et al [14] defined concordance as nonmatching in relation to all the evaluated variables. The exact 95% CIs for the ratios were derived using quantiles of the *F* distribution (Clopper-Pearson intervals) [25].

We analyzed the quality of the information concerning the number of children in the family, number of episodes and agreement of episodes at the family level, and sociodemographic data (gender and date of birth) at the profile level as well as provided information about physician visits, antipyretics, antibiotics, and maximum temperature during the fever episode in the FeverApp registry in comparison with the entries in the physician’s reference records at the level of fever episodes.

### Ethics Approval

The study was conducted in accordance with the guidelines of the Declaration of Helsinki, approved by the Ethics Committee of the University of Witten/Herdecke (protocol code 139/2018 on December 13, 2018) on pseudonymized data collection using an app, and received a positive vote by the data protection service.

## Results

### Overview

**Figure 2:**
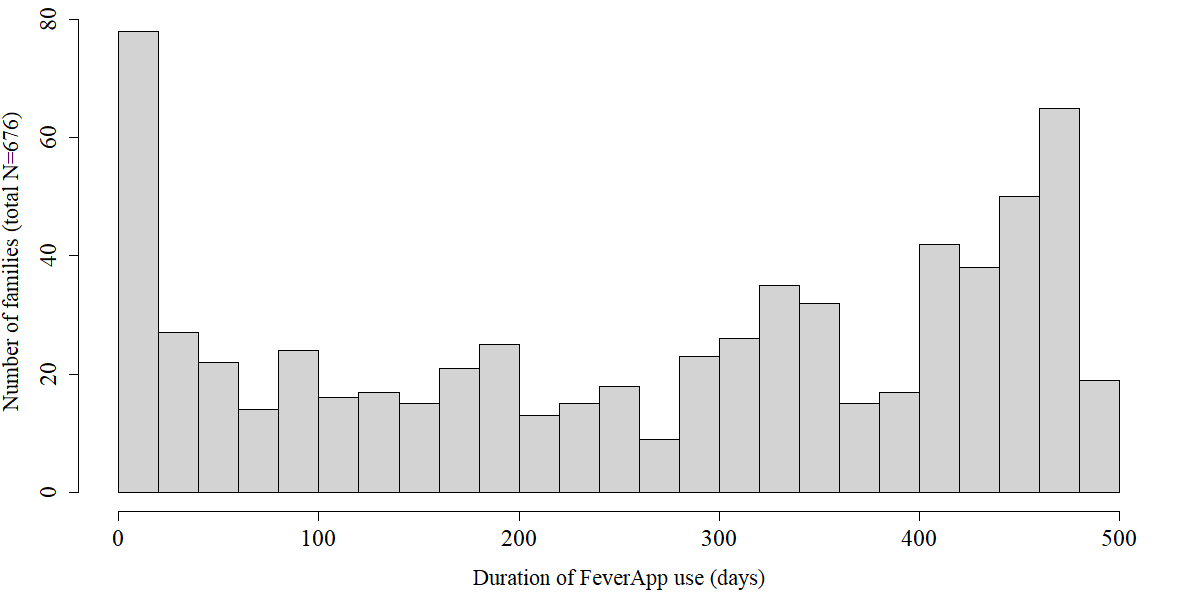
Duration of FeverApp use by 676 participating families.

The results are considered level by level. Naturally, the focus of the analysis is on the level of the fever episodes, which already aggregates several variables over a period. This study considered consecutive enrollment in the 16-month period between September 2019 and December 2020. For each participating family, the duration of FeverApp use varied depending on both the registration date and the need because of fever phases. The median (IQR) time of use of FeverApp by families was 302 (105-423) days, as shown in the histogram (Figure 2).

### Family and Children’s Numbers in Reference Records and in the FeverApp Registry

Consideration of the family level shows that 1273 families with 2009 children signed the participation agreement and received an access code to use the app. In total, there were 3579 patients in the pediatric office during the observation period of 16 months. Therefore, the physician’s office invited 56.13% (2009/3579) of their patients to the FeverApp registry during this time. In comparison, the app-based registry showed that 684 (684/1273, 53.73%) families, with a total of 1047 (1047/2009, 52.12%) profiles, completed the registration process for the app registry during the same period. However, 5 of these families with a total of 6 children could not be identified in the physician’s reference records, probably because of errors in processing the exported EHR comparison data. In addition, 3 registered families did not register any profiles for their children but installed the app. Their profiles in the office records cannot be compared with those in the app-based registry.

Only 24 (24/676, 3.6%) of the remaining 676 (676/684, 98.8%), respectively 679 (679/684, 99.3%) registered families registered more profiles in the app-based registry than in the pediatric office. Of the 676 families, 50 (50/676, 7.4%) did not register all their children in the FeverApp registry. Of the registered families, 602 (89%) registered all their children in FeverApp.

A close look shows that 24 (2.31%) of the remaining 1041 profiles from 676 families belong to persons who are not patients in the office (22 siblings and 2 mothers). Moreover, 5 children have double (synonymous) profiles (confer in the GAMOQ as TMF-1029): this can occur if 2 parents register their children on 2 mobile phones simultaneously due to the time lag of synchronization with the server. Therefore, a comparison of 1012 registry profiles with the records in the pediatric office was possible for a total of 676 families. The word “profiles” in this analysis refers to the profiles of children because all adult profiles (parents) were excluded. There were 3 (3/684, 0.4%) families who installed the app without any profile. Thus, there were 679 families, of which only 676 had a profile.

**Figure 3:**
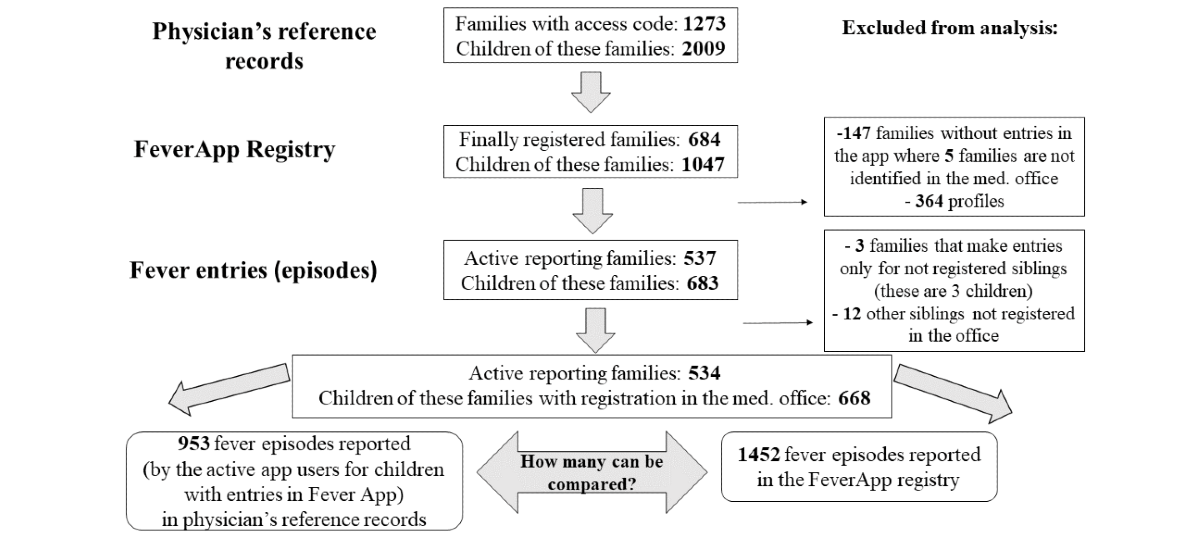
Units of observation at all 3 levels in both data sources.

The 679 families that installed the app reported 1171 fever episodes in the pediatric office. Not all have used FeverApp as a fever diary during the observation period: only 537 (537/684, 78.5%) participating families with 683 children documented 1481 fever episodes in the FeverApp registry. They reported 1038 fever episodes at the same time in the pediatric office. If we exclude 29 episodes of siblings that have no registration in the office’s registry, then 534 families (3 families make entries only for nonregistered children) and 668 children remain with 1452 episodes. In contrast, there were 953 reported episodes for these children in the pediatric office. The flowchart (Figure 3) depicts the process of participation in the FeverApp registry study at all 3 levels and reports the fever episodes that could be used for comparison.

### Recorded Number of Fever Episodes at Family and Child Levels

**Figure 4:**
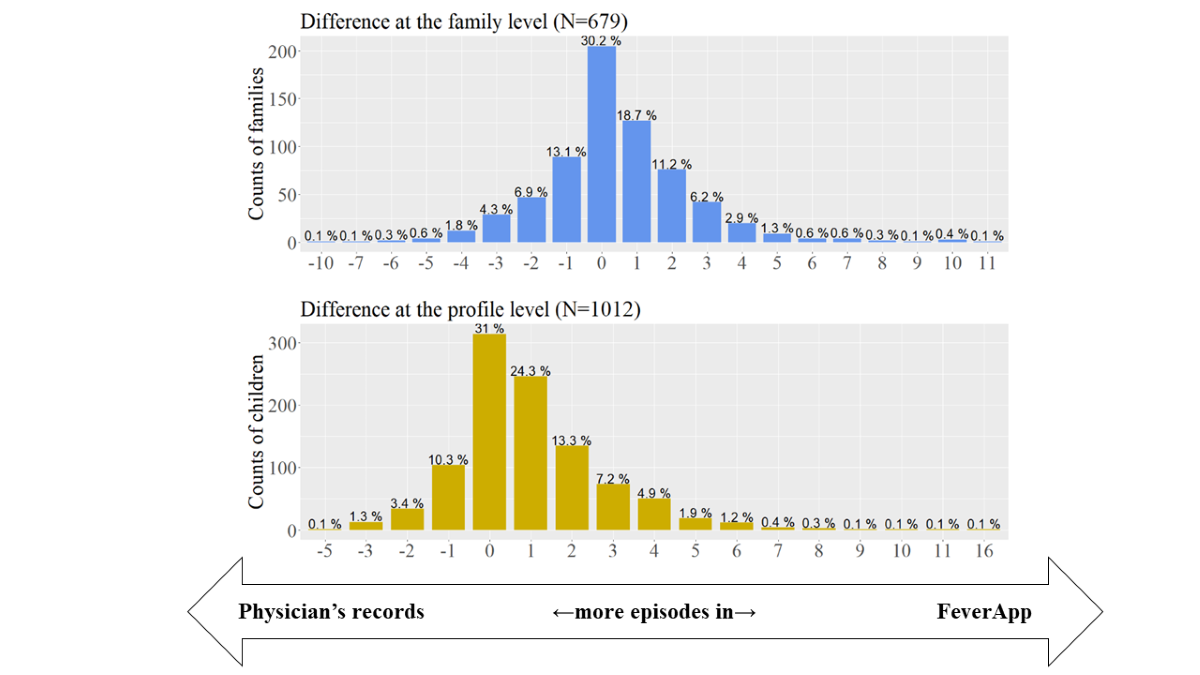
Difference in episode numbers between the app-based registry and reference records.

In the following part of this study, we will look closer at the number of finally comparable episodes, which depends on the observation unit definition. As mentioned, each child in each family can have multiple fever episodes. In Figure 4, the numbers and percentages of registered families (cornflower blue) and registered profiles of children (yellow) with differences in the number of fever episodes between the app-based registry and reference records can be seen on the axis of abscissae. In the axis of ordinates, the absolute and relative frequency of the 1012 children’s profiles from the app, corresponding to 679 registered families, could be seen.

Positive differences indicate that the number of episodes in the app-based registry is greater than the number of episodes in the reference records. Zero indicates that the number of episodes in both sources is equal. It is worth mentioning that 30.2% (205/679) of the families and 31.02% (314/1012) of the children had an equal number of episodes in the app’s and physician’s registries or even had more fever episodes in the app (546/1012, 53.95% children and 289/679, 42.6% families). Therefore, most users do not always contact the physician during the fever episodes of their children.

### Comparison of Children’s Sociodemographic Data

First, completeness and concordance at the second level (Figure 1) were analyzed. In contrast to the app-based parental registry, the EHRs of physicians’ offices consider only patients (profiles) and not complete families. The assignment of persons for comparison is difficult because the identification numbers are different for each data source (sequential number in the reference records in the pediatric office and randomly generated combinations of numbers and letters in the FeverApp registry). Therefore, the family code of FeverApp and children’s gender and date of birth were used to identify comparable profiles. In the following sections, all comparisons are made at the profile level (level 2 according to Figure 1) and not at the family level.

A comparison of entries for gender and date of birth demonstrated that the FeverApp data includes 22 siblings without registration in the office registry and 2 parents. We compared the remaining 1012 (1012/1036, 97.68%) profiles of 676 families (without the 5 synonymous profiles mentioned earlier) based on demographic information. They include 8 nonstatements of the date of birth, 18 errors in the date of birth (n=11, 1.09%), gender (n=6, 0.59%), or both (n=1, 0.10%). The presence of different options for answers for the variable gender (3 in the FeverApp registry and 2 in the physician’s registry) is also a potential cause for disagreements. Most errors in gender (5/6, 83%) occurred in the physician’s registry, and all 19 errors in date of birth occurred in FeverApp, where only month and year of birth are recorded. According to the names in the registered profiles, it can be decided which registry includes incorrect values for gender. There were only 0.69% (7/1012, gender) and 1.19% (12/1012, date of birth) of mismatches between the sources. Therefore, the concordance rates were 99.31% and 98.81%, respectively.

Completeness for gender and date of birth, as expected, reached 100% in the reference records but only 99.2% in the app-based registry for date of birth. This could be because the submission of the month and year of birth was not mandatory in earlier versions of FeverApp.

### Comparison of Fever Episodes

Because of differences in the number of recorded fever episodes at the family and child levels (Figure 4), the analyses of concordance and completeness at the episode level (level 3 according to Figure 1) was more challenging. As stated in Figure 3, there were only 953 reported fever episodes of finally participating families in the FeverApp registry being recorded in physician’s office reference records, whereas approximately 50% (1452/953) more fever episodes were entered by parents in the EMA-based FeverApp registry.

### Comparable Data

**Figure 5:**
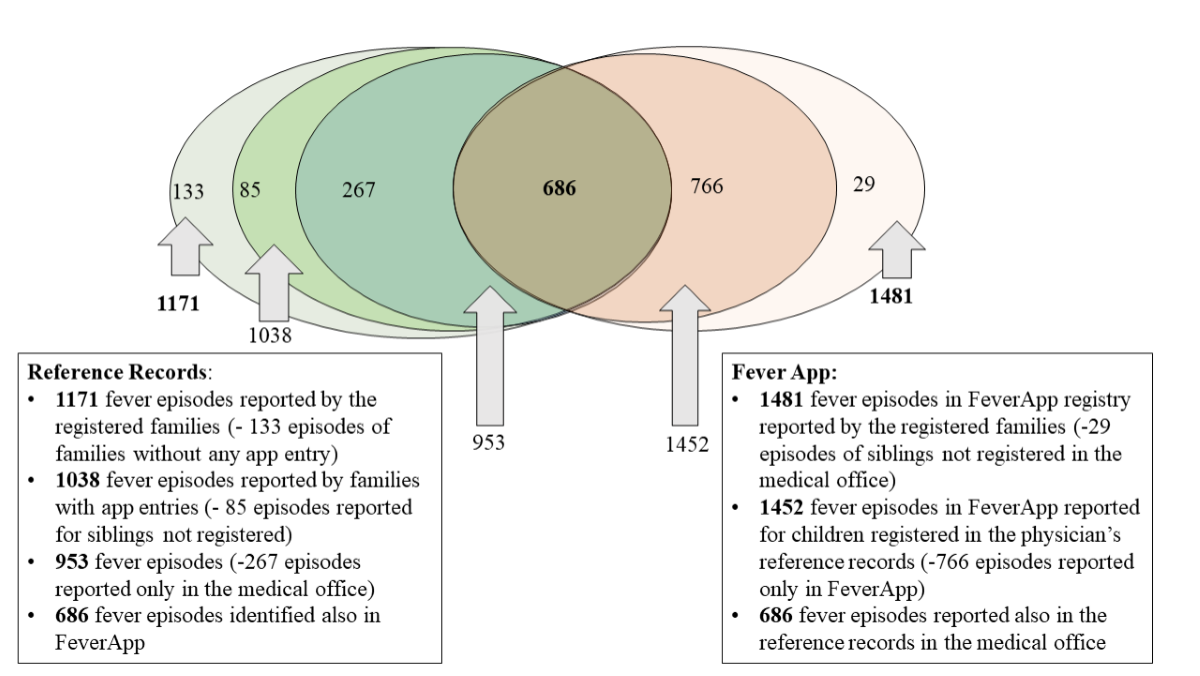
The Venn diagram of fever episodes in the reference records and app-based registry.

To depict comparable fever episodes (on level 2 according to Figure 1) from these 2 sources, a Venn diagram (Figure 5) illustrates the sets of fever episodes in both the reference records in the physician’s office and the central FeverApp registry.

In Figure 5, the 3 green ovals on the left side represent the episodes from the physician’s records as reference. In total, 1171 fever episodes were reported by families with registration in the app; that is, they not only signed the informed consent but also installed the app. Of the 1171 episodes in the office, 133 (11.362%) originated from families without any episode entry in the app. Families with app entries reported the remaining 1038 (88.64%) episodes in the office. As mentioned earlier, 50 (50/537, 9.3%) families did not register all their children, such that 85 (85/1038, 8.19%) fever episodes recorded at the office were from children without profiles in FeverApp. The remaining 953 episodes in the reference records originated from children with profiles in the app. They can be distinguished as 681 (71.5%) acute and 272 (28.5%) past episodes.

The 2 orange ovals on the right side represent the fever episodes from the app-based registry. In total, these were 1481 episodes, with 29 episodes from children who could not be identified in the physician’s records. The remaining 1452 fever episodes from the app registry were from children who could be identified in the physician’s records.

Only 686 episodes in the olive intersection were comparable, where children with profiles and fever episodes in FeverApp also visited the physician’s office after the parents signed the consent to participate. These are 71.9% (686/953) fever episodes from the physician’s records, which originate from children with profiles in the app and 47.25% (686/1452) of fever episodes in the app registry from children who could be identified in the physician’s records. In total, 424 fever episodes in FeverApp were reported as acute and 262 as past in the physician’s reference records. Therefore, 96.3% (262/272) of the past episodes and 62.3% (424/681) of the acute episodes noted in the medical office were also recorded in FeverApp.

Of the 351 families that visited the office owing to an acute fever episode, 37.7% (257/681) of acute episodes were not documented additionally in the registry. Of the 534 families with entries in the app, a similar percentage (183/534, 34.3%) of families refrained from visiting the office during fever episodes but nevertheless documented 338 (338/1452, 23.28%) episodes in FeverApp. These families seem to feel safe using solely FeverApp as support. Additionally, 188 (188/351, 54.4%) families visited the office, but partially refrained to report their episodes (428/1452, 29.48%) in physician’s office.

To calculate the concordance rates, it is sensible to use only the information that can be found in both data sources. Therefore, we compared 686 episodes of 351 families in terms of physician visits, medication, and maximum temperature during the fever episodes.

#### Completeness of Fever Episodes

The completeness of data concerning maximum temperature, physician visits, and medication during fever episodes was analyzed at the level of fever episodes (level 3 according to Figure 1).

Table 1 presents the median, IQR, and total range for the maximum temperature (in ℃) during a fever episode for records from the app registry and for past and acute episodes from the reference records separately.

**Table 1:** Characteristics of the maximum temperature of a fever episode in °C.

	Value, median (IQR)	Total range
FeverApp registry	38.9 (38.3-39.5)	36.2-41.6
Past episodes in the reference records	39.2 (38.9-39.7)	38.0-41.0
Acute episodes in the reference records	39.4 (39.0-39.9)	38.0-42.4

Table 2 summarizes the agreement in FeverApp and reference records. In the analysis, it was assumed that missing answers concerning physician visits and medicaments were equal to negation. In medication, we considered antipyretics and antibiotics separately and no other drugs. The results for FeverApp are presented in the first row: only in 27.55% (400/1452) of the records, parents admitted visiting the physician’s office, and in 30.99% (450/1452) and 3.17% (46/1452) of the episodes, they gave antipyretics and antibiotics, respectively, to their feverish child. In 97.45% (1415/1452) of the episodes, the question about body temperature was answered. The second row presents the answers concerning physician visits, medication, and maximum temperature per fever episode provided in the reference records of the physicians’ office.

The third row presents the subset of all the 1452 episodes entered in FeverApp: 686 fever episodes in the app, which can also be identified in reference records (Figure 5). The fourth row presents 686 fever episodes of the 953 office-registered episodes that can also be identified in the app registry (Figure 5). The comparison of the third and fourths rows shows that the answers concerning medication, physician visits, and maximum temperature per fever episode given in the app and office often differ.

The number of agreements for each of the 4 data elements is shown in the fifth row. The agreement was the lowest regarding the reported maximum temperature and differed between acute (90/424, 21.2% of possible agreements) and past (20/262, 7.6% of possible agreements) reported episodes in the reference records (χ^2^_1_=21.225; *P*<.001).

Table 3 summarizes completeness rates with corresponding 95% CIs for both data sources: the rates are much lower as the usually used DQI benchmark of 95% in both sources, although they are higher in the app-based registry.

Completeness ranges from 11.5% to 65% for the app registry and from 7.6% to 42.6% for the reference source, as shown in the 2 rows in Table 3.

**Table 2:** Response and agreement for submitted physician visits, antipyretics, antibiotics, and temperature in both sources.

		Physician visits			Antipyretics				Antibiotics				Maximum temperature per episode		
		Yes	No		Yes		No		Yes		No		Answered		Not answered
**Episodes in FeverApp (N=1452) and reference records (N=953)**															
	FeverApp episodes, n (%)	400 (27.55)	1052 (72.45)		450 (30.99)		1002 (69.01)		46 (3.17)		1406 (96.83)		1415 (97.45)		37 (2.55)
	Reference records, n (%)	744 (78.1)	209 (21.9)		552 (57.9)		402 (42.1)		90 (9.4)		863 (90.6)		931 (97.7)		22 (2.3)
**Corresponding episodes (n=686)**															
	FeverApp, n (%)	279 (40.7)	407 (59.3)	274 (39.9)		412 (60.1)		32 (4.7)		654 (95.3)		675 (98.4)		11 (1.6)	
	Reference records, n (%)	474 (69.1)	212 (30.9)	418 (60.9)		268 (30.1)		61 (8.9)		625 (91.1)		576 (84.0)		110 (16.0)	
	Agreements between the FeverApp and reference source, n (%)	245 (35.7)	178 (25.9)		234 (34.1)		223 (32.5)		13 (1.9)		606 (88.3)		110 (16.0)		N/A^a^

^a^N/A: not applicable; it is not possible to compare not submitted answers.

**Table 3:** Completeness rates for submitted physician visits, antipyretics, antibiotics, and temperature with corresponding 95% CI.

	Physician visits	Antipyretics	Antibiotics	Maximum temperature in 0.1°C resolution
Completeness rate of app registry in relation to 953 reference records, n/N (%; 95% CI)	423/953 (44.4; 41.2-47.6)	457/953 (48; 44.7-51.2)	619/953 (65; 61.8-68.0)	110/953 (11.5; 9.6-13.7)
Completeness rate of reference records in relation to N=1452 in app registry, n/N (%; 95% CI)	423/1452 (29.13; 26.8-31.5)	457/1452 (31.47; 29.1-33.9)	619/1452 (42.63; 40.1-45.2)	110/1452 (7.58; 6.3-9.1)

#### Concordance of Fever Episodes

**Figure 6:**
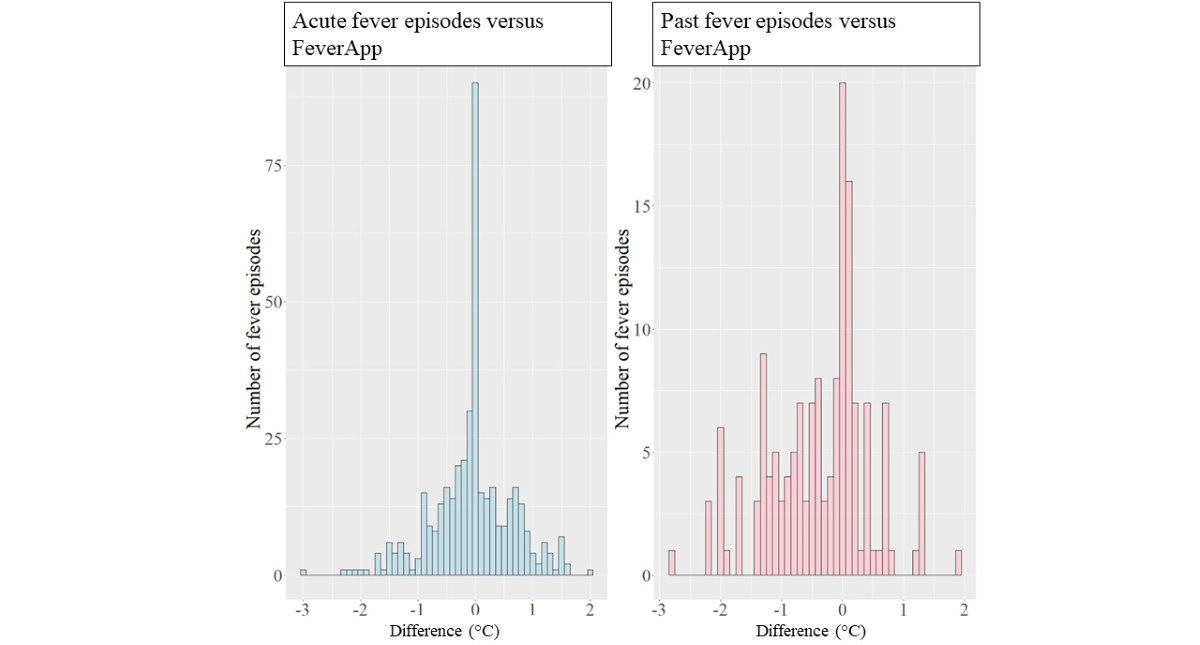
Differences in the maximum temperature between reference records and the app-based registry for 424 acute and 262 past fever episodes.

Table 4 summarizes all concordance values with corresponding 95% CI and frequencies of agreement at the episode level (level 3 according to Figure 1). The concordance rates were varied: 90.2% in terms of antibiotics, 66.6% in terms of antipyretics, 61.7% in terms of physician visits, and 16% in terms of maximum temperature. The lowest rate of agreement was observed for the maximum temperature per episode. This depends on the resolution of the metric measure at a temperature of 0.1 °C. With less subtle resolution, higher agreement rates are possible. Therefore, in Figure 6, we present the histograms of the temperature differences between acute and past fever episodes. Differences between the values from the FeverApp records and those from the reference office records were in the range of −2 °C to 3 °C. Positive differences indicated that the submitted maximum temperature per fever episode in the app records was higher than that in the reference records. The IQR for acute fever episodes lies within the range of −0.3 °C to 0.4 °C. The IQR is wider for past fever episodes: from −0.1 °C to 0.9 °C (Figure 6). The Mann-Whitney test showed a significant difference between acute and past fever episodes (*W*=38,854; *P*<.001).

**Table 4:** Concordance rates of comparable data elements.

	Physician visits (N=686)	Antipyretics (N=686)	Antibiotics (N=686)	Maximum temperature in 0.1 °C resolution (N=686)
Agreement, n	423	457	619	110^a^
Concordance rate (%)	61.7	66.6	90.2	16.0
95% CI	57.9-65.3	63.0-70.0	85.7-90.6	13.4-19.0

^a^Expecting exact agreement, see Figure 6.

## Discussion

### Principal Findings

As part of a publicly funded model registry initiative [4], 6 registries aim to implement several DQIs for drawing comparison between very different registries. The 2 presented DQIs, completeness and concordance, cover 2 of the 5 dimensions according to Weiskopf and Weng [10]. In contrast, according to the GAMOQ [14], these 2 DQIs (concordance and completeness) belong to the dimension of trueness. These dimensions seem to be diversely understood in the comparison of different registries, resulting in interpretation difficulties [26]. Therefore, because it is especially important for an EMA-based registry, we herewith contribute to shedding light on an example with a multiple clustered observation unit.

This study has provided several new insights into research on the possibilities in ambulatory pediatric care and demonstrates the use of DQIs. First, it demonstrates that in all analyses of clustered observation units, the cluster level must be mentioned and considered in separate analyses. Second, although the cooperating pediatricians purposefully and systematically collected data to create a reference for the FeverApp registry, with a high motivation to assure high quality of gathered office data, the records in the pediatric office were less complete than the parental recordings in FeverApp. This finding was surprising to the authors and reversed their perspective: in many situations, such as the present example of comparing FeverApp to office records or even to extra office-based febrile history records, app-based EMA is of higher quality. Therefore, medical practice records should not be seen as the gold standard in comparison with the app-based approach.

The data element gender was the most complete, with only few disagreements due to mistakes. The question about date of birth was not mandatory until the release of version 1.7.2 of the app in October 2020. The data on gender and date of birth in the FeverApp profiles, together with the physician’s records, shows high concordance (>98%) and even perfect completeness because of the obligation to fill the selection fields of gender and date of birth.

A comparison of the number of children and the number of episodes at the family level (679 families) between both data sources gives only a limited view on complete values. Although families are seen as observation units in the FeverApp registry, it is essential to analyze data quality at lower levels. To avoid biases, result profiles and even single fever episodes must be considered. A comparison of these levels seems to be much more informative concerning the real quality of data. For example, registered families can use the app for siblings who are not patients of the pediatrician’s office. Hence, a simple comparison of the number of fever episodes per family seems to differ strongly, without clarity as to whether they belong to the same patient. The number of episodes per family may be higher than the number of fever episodes in the reference records for children registered without their siblings. Alternatively, the information does not differ at all; for example, the number of fever episodes seems to be equal between both sources because it is not guaranteed that parents submit information about the same child or the same episode. Therefore, it is essential to compare the fever episodes of each registered child based on the information available in both sources: the date of physician visit, medication with antipyretics and antibiotics, and maximum temperature.

We observed a descent of concordance values for nonmandatory elements: parents often do not submit information concerning physician visits in the app during an acute fever episode. The relatively high grade of agreement for antibiotics could be caused by the rare prescription of antibiotics in this pediatric office during the observation period. In addition, information about typical antipyretic medications may not be submitted to the app-based registry or to the physician’s office. Without mandatory entries, high completeness rates of 95% are quite illusory. Hence, thresholds depend on the circumstances of data collection and cannot be generalized. High DQI values may be easily produced through the analysis of accumulated data level. A low level could occur because of families that do not consider the documentation of medication as important and, hence, mandatory. Therefore, neither complete nor concordant data capture should be expected. On the other hand, temperature is very often only roughly recorded in physicians’ offices.

In contrast to clinical research, there are fewer mandatory fields in public health research, and the kinds and levels of these variables are much more diverse. To overcome this issue other view in public health or even EMA as a possible solution. If research circumstances allow, we suggest that each person collecting data define their own mandatory fields according to their needs.

In app research, a short duration of use is often expected and may produce some kind of proinnovation bias; that is, in the beginning, the app may be used more often. Figure 2 depicts clearly that the duration of use was not skewed, and a remarkable period of app use was confirmed.

### Limitations

The approach of using reference records for comparison, regarding completeness and concordance, has limitations. This comparison is only possible for the observation period between informed consent and last attendance at the pediatric office. This may be a reason for the approximately 50% higher number of episodes in the FeverApp registry than that in the reference records. The extent may be even greater because of the pandemic [5]. However, children have mandatory office consultations because of vaccinations and examinations; therefore, we assumed no influence on the total number of fever events. Nevertheless, the number of acute and past fever events may differ and may shift the numbers in Figure 5. However, without a nonpandemic observation period, further conclusions were impossible.

Nevertheless, the achieved EMA quality in direct comparison with that of professionally acquired data is extraordinary. For some discrepancies, it was not possible to verify which of the data sources was correct. Theoretically, it is possible that both data sources may contain errors in the same direction, which would render such errors unnoticeable during comparison.

We validated the data for 1 office with the highest number of participants and very accurate documentation by the medical personnel in the office, and it is possible that the pediatrician in the office has a positive motivational influence on the users of the app. The extension of the study to other participating offices is desirable and would increase the significance of the study but is difficult to implement because of the high effort required from the medical personnel in the offices.

Of course, data quality analyses can be extended in various directions; for example, extension to further dimensions in structure and integrity according to Donabedian [19]. Data quality statements and investigations are still a stepchild in research, and the well-known FAIR (Findable, Accessible, Interoperable, Reusable) principles on data could be extended by their quality, as could FAIR-Q (Findable, Accessible, Interoperable, Reusable and Quality) [26].

### Comparison With Other Studies

According to other studies, users show a common behavior: participating parents kept fever diaries on paper [3] or used a mobile app [2] during a certain period, and many parents stopped filling out the diaries after their child recovered [3] or forgot to answer the questions because of different external factors (eg, stress) [2].

The data quality of mandatory data elements in FeverApp is comparable with a study from 1993 [27], where entries were done by medical personnel. The results of Kenny et al [28] show that the input of date of birth has a high potential for mistakes; therefore, the quality of this data element should be assured.

Data quality in clinical registers is reported as generally high [29,30]. There are many possibilities to assess the completeness of data in clinical registries: source data verification, comparison of established epidemiological measures such as incidence rates, cumulative incidence curves, and incidence mortality ratios with external databases [15]. However, these methods are not appropriate for the app-based registry FeverApp because of the lack of a data source owing to momentary assessment. In addition, epidemiological measures for fever are not available because it is only a symptom of heterogeneous diseases. Therefore, lower thresholds for existing DQIs are required in this case.

Recently, Schmidt et al [18] presented a set of DQIs developed specifically for the assessment of data quality in health research. A possible step forward could be a complete evaluation of the data sets from the FeverApp with this extension of the GAMOQ framework. Furthermore, Kapsner et al [31] developed a tool for EHR data quality assessment in clinical research, which can be used for multidimensional data and may be also used for the data from the FeverApp registry.

Kenny et al [28] suggested a possibility to avoid comparison with other sources. This technique suggests validation relaxation for data collected via mobile devices: this is the intentional omission of electronic data validation features for selected questions to allow for data recording errors to be committed, detected, and monitored.

Doherty et al [32] mentioned the data quality issue of EMA data in their work. Review studies [33,34] show in their analyses that compliance and completeness rates of EMA studies are under a desirable level of 80%, but they provide no uniform conclusion regarding the reason for this. Ono et al [34] concluded that the duration of the study influences the completeness rates of the EMA data, whereas Jones et al [33] and Wen et al [35] did not find any significant influence of duration. Nevertheless, Yang et al [36] showed that completeness rates in the daily EMA study decreased after 5 days of use.

Concordance is seldom a major focus in EMA studies with mobile phones. Our values of concordance are comparable with that of Olson et al [2] but lower than those in the study by Hopper et al [37] from 2006, where the investigation of the completeness and concordance of the ActiWatch device data was a part of an EMA study concerning drug intake.

Nowadays, public health researchers must deal more and more with not only EMA data but also EHRs in general, which are in itself limited in completeness, as shown recently by Weiskopf et al [38]. This study explains this issue in detail for 2 very important DQIs as part of an elaborate framework.

### Conclusions

Despite purposeful and systematic data collection by pediatricians, the parental real-time recordings in the FeverApp registry were more complete. Public health data, especially parental EMA data, cannot be easily compared with the same thresholds of clinical registries. Especially data completeness depends on the obligation to answer. For the comparison of quality, the indicator’s obligation, source, level, and kind of variable have to be considered carefully.

Data completeness in registries based on optional self-documentation is not comparable with that in clinical registries by medical professionals (eg, for cancer), where all data elements are mandatory. A further conclusion is that although families are the main observation units, it is necessary to analyze more specific levels (profiles and fever episodes) to avoid incorrect conclusions concerning data quality aspects such as completeness. Test entries or omissions of data in this app-based registry were not seen as shortcomings because of its educational approach. Educated parents may use the app less frequently over time and visit the pediatrician only if necessary. This behavior must be taken into account during assessing and improving the data quality of app-based registries.

In direct comparison with a highly motivated professional office, the EMA-based registry shows how much data, and hence the quality indicators, depend on the acquisition method. It has been shown that EMA by parents can supplement ambulatory care, especially during the pandemic. This study is particularly interesting in light of the fact that mobile apps will have a much greater presence in patient care in the future.

## Appendix

Multimedia Appendix 1 Fever variables in doctor's office reference records.

Multimedia Appendix 2 Data files in SPSS format.

## Abbreviations

DQI: data quality indicator
EHR: electronic health record
EMA: ecological momentary assessment
FAIR: Findable, Accessible, Interoperable, Reusable
FAIR-Q: Findable, Accessible, Interoperable, Reusable and Quality
GAMOQ: Guidelines for the Adaptive Management of Data Quality for Cohort Studies and Registers
TMF: The Technology and Methodology Platform for Networked Methodological Medical Research

## Data Availability

The data presented in this study are available in SPSS formats as supplementary material Multimedia Appendix 2.

## References

[1] Martin D, Wachtmeister J, Ludwigs K, Jenetzky E (2020). The FeverApp registry - ecological momentary assessment (EMA) of fever management in families regarding conformity to up-to-date recommendations. BMC Med Inform Decis Mak.

[2] Olson D, Lamb M, Lopez MR, Colborn K, Paniagua-Avila A, Zacarias A, Zambrano-Perilla R, Rodríguez-Castro SR, Cordon-Rosales C, Asturias EJ (2017). Performance of a mobile phone app-based participatory syndromic surveillance system for acute febrile illness and acute gastroenteritis in rural Guatemala. J Med Internet Res.

[3] Kool M, Elshout G, Moll HA, Koes BW, van der Wouden JC, Berger MY (2013). Duration of fever and course of symptoms in young febrile children presenting with uncomplicated illness. J Am Board Fam Med.

[4] (2016). Model registers - realization phase. Bundesministerium fur Bildung und Forschung.

[5] Jenetzky E, Schwarz S, Fingerhut I, Kerdar SH, Gwiasda M, Rathjens L, Kulikova O, Martin D (2021). The FeverApp Registry - a way to empower parents through their own documentation to a graduated decision. Gesundheitswesen.

[6] Davis T (2013). NICE guideline: feverish illness in children--assessment and initial management in children younger than 5 years. Arch Dis Child Educ Pract Ed.

[7] Larson SJ, Dunn AJ (2001). Behavioral effects of cytokines. Brain Behav Immun.

[8] Sullivan JE, Farrar HC, Section on Clinical PharmacologyTherapeutics, Committee on Drugs (2011). Fever and antipyretic use in children. Pediatrics.

[9] Corrard F, Copin C, Wollner A, Elbez A, Derkx V, Bechet S, Levy C, Boucherat M, Cohen R (2017). Sickness behavior in feverish children is independent of the severity of fever. An observational, multicenter study. PLoS One.

[10] Weiskopf NG, Weng C (2013). Methods and dimensions of electronic health record data quality assessment: enabling reuse for clinical research. J Am Med Inform Assoc.

[11] Kahn MG, Raebel MA, Glanz JM, Riedlinger K, Steiner JF (2012). A pragmatic framework for single-site and multisite data quality assessment in electronic health record-based clinical research. Med Care.

[12] Weiskopf NG, Bakken S, Hripcsak G, Weng C (2017). A data quality assessment guideline for electronic health record data reuse. EGEMS (Wash DC).

[13] Lee K, Weiskopf N, Pathak J (2017). A framework for data quality assessment in clinical research datasets. AMIA Annu Symp Proc.

[14] Nonnemacher M, Nasseh D, Stausberg J (2014). Datenqualität in der medizinischen Forschung.

[15] Jacke CO, Kalder M, Wagner U, Albert US (2012). Valid comparisons and decisions based on clinical registers and population based cohort studies: assessing the accuracy, completeness and epidemiological relevance of a breast cancer query database. BMC Res Notes.

[16] Jacke CO, Kalder M, Koller M, Wagner U, Albert US (2012). Systematic assessment and improvement of medical data quality. Bundesgesundheitsblatt Gesundheitsforschung Gesundheitsschutz.

[17] Frese J, Gode A, Heinrichs G, Will A, Schulz A (2019). Validating a transnational fracture treatment registry using a standardized method. BMC Med Res Methodol.

[18] Schmidt CO, Struckmann S, Enzenbach C, Reineke A, Stausberg J, Damerow S, Huebner M, Schmidt B, Sauerbrei W, Richter A (2021). Facilitating harmonized data quality assessments. A data quality framework for observational health research data collections with software implementations in R. BMC Med Res Methodol.

[19] Donabedian A (1980). Explorations in quality assessment and monitoring Vol. 1. The Definition of Quality and Approaches to Its Assessment.

[20] Aronsky D, Haug PJ (2000). Assessing the quality of clinical data in a computer-based record for calculating the pneumonia severity index. J Am Med Inform Assoc.

[21] Shiffman S, Stone AA, Hufford MR (2008). Ecological momentary assessment. Annu Rev Clin Psychol.

[22] Jenetzky E (2020). Data structure of FeverApp registry. FeverApp.

[23] R Core Team (2020). R: A Language and Environment for Statistical Computing.

[24] Wickham H (2016). Ggplot2 Elegant Graphics for Data Analysis.

[25] Rinne H (2008). Taschenbuch der Statistik.

[26] Stausberg J, Harkener S, Jenetzky E, Jersch P, Martin D, Rupp R, Schönthaler M (2022). FAIR and quality assured data - the use case of trueness. Stud Health Technol Inform.

[27] Schouten L, Jager J, van den Brandt P (1993). Quality of cancer registry data: a comparison of data provided by clinicians with those of registration personnel. Br J Cancer.

[28] Kenny A, Gordon N, Griffiths T, Kraemer JD, Siedner MJ (2017). Validation relaxation: a quality assurance strategy for electronic data collection. J Med Internet Res.

[29] Schmidtmann I, Blettner M (2018). How do cancer registries in Europe estimate completeness of registration?. Methods Inf Med.

[30] Meyer AC, Hedström M, Modig K (2020). The Swedish Hip Fracture Register and National Patient Register were valuable for research on hip fractures: comparison of two registers. J Clin Epidemiol.

[31] Kapsner LA, Kampf MO, Seuchter SA, Kamdje-Wabo G, Gradinger T, Ganslandt T, Mate S, Gruendner J, Kraska D, Prokosch H (2019). Moving towards an EHR data quality framework: the MIRACUM approach. Stud Health Technol Inform.

[32] Doherty K, Balaskas A, Doherty G (2020). The design of ecological momentary assessment technologies. Interact Comput.

[33] Jones A, Remmerswaal D, Verveer I, Robinson E, Franken IH, Wen CK, Field M (2019). Compliance with ecological momentary assessment protocols in substance users: a meta-analysis. Addiction.

[34] Ono M, Schneider S, Junghaenel DU, Stone AA (2019). What affects the completion of ecological momentary assessments in chronic pain research? An individual patient data meta-analysis. J Med Internet Res.

[35] Wen CK, Schneider S, Stone AA, Spruijt-Metz D (2017). Compliance with mobile ecological momentary assessment protocols in children and adolescents: a systematic review and meta-analysis. J Med Internet Res.

[36] Yang YS, Ryu GW, Choi M (2019). Factors associated with daily completion rates in a smartphone-based ecological momentary assessment study. Healthc Inform Res.

[37] Hopper JW, Su Z, Looby AR, Ryan ET, Penetar DM, Palmer CM, Lukas SE (2006). Incidence and patterns of polydrug use and craving for ecstasy in regular ecstasy users: an ecological momentary assessment study. Drug Alcohol Depend.

[38] Weiskopf NG, Cohen AM, Hannan J, Jarmon T, Dorr DA (2019). Towards augmenting structured EHR data: a comparison of manual chart review and patient self-report. AMIA Annu Symp Proc.

