# Supplementary material for: Data Completeness and Concordance in the FeverApp Registry: Comparative Study
Source: JMIR Pediatr Parent. 2022 Nov 2;5(4):e35510. doi: 10.2196/35510 (PMC9669892; doi:10.2196/35510)
Supplement: Multimedia Appendix 1 [file pediatrics_v5i4e35510_app1.docx]

Presentation currently with (start at 1.) or without (start at 4.) fever

## (= at least once "fever" according to parents and/or temp. >38,5°C within the last 24 h)

1. How many days does fever exist?

### Selection: 1 (within the last 24 h) / 2 / 3 / 4 / 5 / [entry number].

1. What was the maximum temperature measured?

Selection: [entry maximum temperature] / don't know

1. Were medications given to reduce fever?

Selection: no / paracetamol / ibuprofen / paracetamol + ibuprofen switching / other / don't know

1. Have there been any febrile episodes in your child since the last presentation that we do not know about yet?

Selection: 0 / 1 / 2 / [entry number] / don't know.

4.1 What was the maximum temperature measured?

Selection: [entry maximum temperature] / don't know

- 1. Was medication given to reduce fever?

Selection: no / paracetamol / ibuprofen / paracetamol + ibupr. switching / other / don't know

4.3 Was a physician consulted (also by telephone or digitally)?

Selection: yes / no / don't know

1. Do you log fever phases?

Selection: no / yes, on paper / yes, with an app / yes, with the FeverApp.
